# Work‐System Factors Associated With Patient Safety Nursing Activities in Integrated Nursing Care Service Wards of Nonaccredited Small‐ and Medium‐Sized Hospitals in Korea: A Cross‐Sectional Study With Exploratory Quantile Regression

**DOI:** 10.1155/jonm/9403660

**Published:** 2026-11-09

**Authors:** Yeong Im Kim, Hye Jin Chong

**Affiliations:** ^1^ Suncheon Hana Hospital Suncheon-Si Jeonnam Gwangju South Korea; ^2^ Department of Nursing Sunchon National University Suncheon-Si, Jeonnam Gwangju South Korea sunchon.ac.kr

**Keywords:** ethical leadership, integrated nursing care service, nursing management, nursing skill mix, patient safety

## Abstract

**Background:**

Patient safety nursing activities are associated with nurses’ safety‐related capabilities and organizational and task‐related work conditions. However, evidence integrating these factors within a work‐system framework in nonaccredited small‐ and medium‐sized hospitals remains limited.

**Aim:**

To examine associations between selected person‐, organizational‐, and task‐related factors and patient safety nursing activities and explore patterns across conditional quantiles using a Systems Engineering Initiative for Patient Safety (SEIPS)–informed framework.

**Methods:**

A cross‐sectional survey was conducted with 218 nurses. Ordinary least squares (OLS) and quantile regression models were fitted at the 10th, 25th, 50th, 75th, and 85th percentiles. Quantile estimates used 2000 pairs‐bootstrap resamples. Coefficient equality across quantiles was tested with Holm adjustment, and fractional logit was used as a bounded‐outcome sensitivity analysis.

**Results:**

Patient safety nursing activity scores were concentrated near the upper boundary (mean = 4.46); 13.8% scored 5.00% and 39.0% scored ≥ 4.75. In OLS analysis, safety control, near‐miss reporting intention, ethical leadership, and non‐nursing task performance were positively associated with patient safety nursing activities, whereas organizational communication and shift‐based nursing skill mix were not. Although point estimates varied across quantiles, no coefficient‐equality test remained significant after Holm adjustment (smallest adjusted *p* = 0.060). Fractional‐logit results were similar.

**Conclusion:**

Patient safety nursing activity scores were high but varied across the score distribution. Safety control showed the clearest exploratory distributional pattern, with larger coefficient estimates at lower‐to‐middle conditional quantiles.

**Implications for Nursing Management:**

Considering both average patient safety nursing activity levels and the underlying score distribution may provide nurse managers with a more complete understanding of patient safety nursing practice. Safety control, reporting readiness, and ethical leadership represent relevant areas for organizational attention.

## 1. Introduction

Patient safety remains a central concern in nursing management because preventable harm is driven not only by individual vigilance but also by staffing, leadership, communication, work allocation, and organizational safety systems [1, 2]. The World Health Organization Global Patient Safety Action Plan 2021–2030 emphasizes governance, safety culture, reporting and learning systems, health worker education, and patient and family engagement as core strategies for reducing avoidable harm [2]. In South Korea, patient safety is supported through national reporting and learning systems and healthcare accreditation mechanisms. Continued attention to patient safety remains important across clinical settings.

Integrated nursing care service wards in nonaccredited small‐ and medium‐sized hospitals provide an important context for examining patient safety nursing activities. Previous studies in integrated nursing care settings have examined non‐nursing tasks, organizational communication, and nursing skill mix separately [3–6], but evidence integrating these domains within a single work‐system framework remains limited. In these wards, registered nurses and nursing assistants jointly provide comprehensive inpatient care [6]. Patient safety nursing activities can therefore be examined in relation to nurses’ safety‐related capabilities [7, 8], leadership [9], organizational communication [4], and task and staffing conditions [3, 5, 6], as organized by the SEIPS framework [9, 10].

Patient safety nursing activities include routine safety‐related nursing behaviors, such as accurate patient identification, communication among healthcare professionals, safe medication management, procedure verification, infection prevention, and fall prevention [2, 11]. These activities are shaped by nurses’ individual safety capacity and by organizational and task‐related work conditions, including leadership, communication, workload, task allocation, and staffing [9, 12]. Therefore, patient safety nursing activities should be understood as work‐system outcomes rather than isolated individual behaviors.

The Systems Engineering Initiative for Patient Safety (SEIPS) framework provides a useful basis for organizing these multilevel influences by conceptualizing patient safety outcomes as the result of interactions among persons, tasks, tools and technology, organizations, and environments [9, 10]. In this study, safety control and near‐miss reporting intention were classified as person‐level factors, ethical leadership and organizational communication as organizational‐level factors, and non‐nursing task performance and nursing skill mix as task‐level factors. In this study, the SEIPS framework was used to organize the selected variables into person‐, organizational‐, and task‐related domains. The conceptual framework is presented in Figure 1.

**FIGURE 1:**
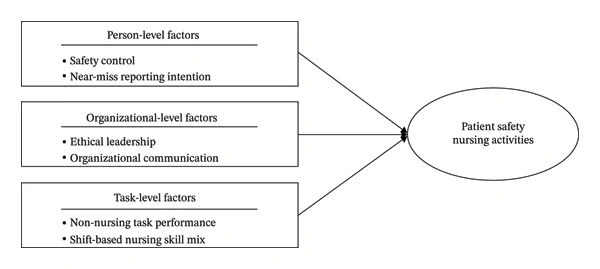
Conceptual framework outlining factors associated with patient safety nursing activities, based on the Systems Engineering Initiative for Patient Safety framework [13] and Holden et al. [14]. Note: Nursing skill mix was operationalized as the shift‐based proportion of registered nurses among registered nurses and nursing assistants actually working across day, evening, and night shifts during the previous month.

Previous studies have identified associations of patient safety nursing activities with safety‐related capabilities, organizational leadership and communication, non‐nursing tasks, and nursing skill mix [3–6, 8]. Most of this evidence has been generated using mean‐based analyses or by examining selected factors separately. Consequently, it remains unclear whether their adjusted associations are consistent across the conditional distribution of patient safety nursing activity scores.

Guided by the SEIPS framework [9, 10], this study simultaneously examined selected person‐, organizational‐, and task‐related factors. Quantile regression was used as an exploratory complement to conditional‐mean analysis to examine coefficient patterns across selected conditional quantiles [13, 14].

Therefore, this study aimed to (1) describe patient safety nursing activities and selected person‐, organizational‐, and task‐related factors; (2) compare these variables according to patient safety incident experience and awareness of near‐miss and patient safety incident reporting procedures; (3) estimate their overall adjusted associations with patient safety nursing activities; and (4) explore whether the corresponding coefficients varied across the 10th, 25th, 50th, 75th, and 85th conditional quantiles.

## 2. Materials and Methods

### 2.1. Design and Participants

This cross‐sectional study examined factors associated with patient safety nursing activities among clinical nurses and was reported in accordance with the Strengthening the Reporting of Observational Studies in Epidemiology guidelines.

Nonaccredited hospitals were defined as hospitals that had not obtained official healthcare accreditation from the Korea Institute for Healthcare Accreditation at the time of data collection. The study hospitals were small‐ and medium‐sized institutions with 200 beds or fewer. All participating hospitals were nonaccredited at the time of data collection; therefore, accreditation status served as a sampling criterion rather than an analytic exposure. Integrated nursing care service wards were defined as Korean nurse‐led inpatient wards in which nurses and nursing assistants deliver comprehensive inpatient care without routine bedside caregiving from family members or privately hired caregivers. The study population included nurses who met the following inclusion criteria: (1) registered nurses providing direct patient care in integrated nursing care service wards; (2) had worked in their current ward for at least 6 months to ensure adequate exposure to the ward environment and patient safety practices; and (3) agreed to participate in the study. Nurses in managerial or administrative positions without routine patient contact were excluded.

Participants were recruited from 10 hospitals across two metropolitan and provincial regions in South Korea. The required sample size was estimated using G∗Power 3.1.9.7 for a six‐predictor OLS regression model. Assuming a medium effect size (*f* ^2^ = 0.15), a two‐sided significance level of 0.05, and a power of 0.90, the minimum required sample size was 123 [15]. Allowing for a 20% attrition rate, 224 nurses were recruited. All 224 questionnaires were returned; after six incomplete questionnaires were excluded, 218 were included in the final analysis, yielding a usable‐response rate of 97.3%. Because the calculation did not assess power for quantile‐specific coefficients or cross‐quantile contrasts, the quantile analyses were treated as exploratory.

### 2.2. Data Collection

Data were collected from March to April 2025 using a self‐administered questionnaire. To improve accessibility and response rates, both online and paper‐based surveys were used. The online survey was administered through Google Forms, whereas paper questionnaires were distributed in person at participating hospitals. Before data collection, approval was obtained from the hospital administrators and nursing departments. Eligible nurses received written information explaining the study purpose, procedures, voluntary participation, and confidentiality safeguards. For the online survey, participants accessed the questionnaire through a secure link and provided electronic informed consent before participation. For the paper‐based survey, written informed consent was obtained before questionnaire completion. Completed paper questionnaires were returned in sealed envelopes to maintain anonymity, and online responses were stored in a password‐protected database accessible only to the research team. Data from both survey modes were combined into a single dataset for analysis.

## 3. Measures

### 3.1. Demographic and Job‐Related Characteristics

The survey collected demographic and job‐related data, including age, gender, marital status, educational attainment, work experience, and hospital bed size. For analysis, these variables were categorized as follows: age (≤ 29, 30–39, or ≥ 40 years), gender (female or male), marital status (single or married), educational attainment (associate, bachelor’s, or graduate degree), work experience (< 1, 1 to < 5, 5 to < 10, or ≥ 10 years), and number of hospital beds (< 100, 100–149, or ≥ 150). Experience with safety incidents in the past year and awareness of near‐miss and patient safety incident reporting procedures were also assessed as yes/no variables.

### 3.2. Patient Safety Nursing Activities

Patient safety nursing activities were measured using a 24‐item instrument developed and validated by Park and Kim [11], based on national healthcare accreditation standards and the International Patient Safety Goals. The instrument assesses six core domains of safety‐related nursing practice: accurate patient identification, communication among healthcare professionals, high‐risk medication management, verification of correct procedures, infection prevention, and fall prevention, which represent key care processes directly linked to patient safety outcomes in clinical settings. Each item was rated on a 5‐point Likert scale ranging from 1 (“never”) to 5 (“always”), with higher scores indicating greater engagement in patient safety nursing activities. The tool has been widely used in Korean nursing research to assess safety‐related clinical performance and has shown strong construct validity (*χ* ^2^ = 375.35, GFI = 0.92, CFI = 0.94, RMSEA = 0.06) and internal consistency reliability (Cronbach’s *α* = 0.89 in the original study; *α* = 0.95 in this study) [11].

### 3.3. Safety Control

Safety control was assessed with a 7‐item Safety Control Scale originally developed by Anderson et al. [16] to measure nurses’ perceived capacity to control and manage patient safety risks in clinical practice. The construct reflects individuals’ belief that their actions can meaningfully influence safety outcomes and serves as a critical psychological resource for proactive safety behaviors. Each item was rated on a 5‐point Likert scale ranging from 1 (“strongly disagree”) to 5 (“strongly agree”), with higher scores indicating greater perceived safety control. The Safety Control Scale has been validated in Korean hospital settings, showing acceptable reliability (Cronbach’s *α* = 0.84) in Jeong’s [17] study and strong psychometric properties in subsequent applications. In the present study, internal consistency reliability was high (Cronbach’s *α* = 0.89).

### 3.4. Near‐Miss Reporting Intention

Near‐miss reporting intention was measured using a 4‐item instrument revised by [18] based on previous measures of patient safety culture and medical‐error reporting [7]. The instrument assesses nurses’ willingness to report incidents that could have caused patient harm but did not. Items were rated on a 5‐point Likert scale (1 = “strongly disagree,” 5 = “strongly agree”), with higher scores indicating stronger reporting intention. Near‐miss reporting is recognized as a key component of organizational learning and safety culture, helping identify latent system risks before adverse events occur [1, 7]. The scale showed acceptable internal consistency in this study (Cronbach’s *α* = 0.82).

### 3.5. Ethical Leadership

Ethical leadership was assessed using the Ethical Leadership Scale (ELS), developed by Brown et al. [19] and translated into Korean by Kim and Jeong [20]. This 10‐item instrument assesses nurses’ perceptions of their immediate supervisors’ ethical conduct, fairness, integrity, and modeling of appropriate behavior. Items were rated on a 5‐point Likert scale, with higher scores indicating stronger perceived ethical leadership. Previous research has linked ethical leadership with patient safety culture in healthcare organizations [21]. The ELS demonstrated excellent internal consistency (Cronbach’s *α* = 0.97) in this study.

### 3.6. Organizational Communication

Organizational communication was assessed using a 23‐item scale adapted for the nursing context by Oh and Yi [22]. The instrument measures the effectiveness of formal (upward, downward, horizontal) and informal communication processes within nursing organizations, including information sharing, feedback, and clarity of communication channels. Each item was rated on a 5‐point Likert scale, with higher scores indicating more effective organizational communication. Effective organizational communication has been associated with teamwork and patient safety outcomes in clinical settings [23]. Internal consistency reliability for this scale was excellent (Cronbach’s *α* = 0.96).

### 3.7. Non‐Nursing Task Performance

Non‐nursing task performance was assessed with a 9‐item instrument developed by Bruyneel et al. [24] and adapted into Korean by Park and Hwang [6]. The scale measures how often nurses perform ancillary or administrative tasks that do not require professional nursing competencies (e.g., patient transport, clerical work, tray delivery). Responses were scored on a 3‐point scale (0 = “never,” 1 = “occasionally,” 2 = “frequently”), with higher scores indicating more frequent non‐nursing task performance. Excessive involvement in such tasks is associated with increased workload, less direct patient care time, and potentially adverse safety outcomes [24]. In this study, internal consistency reliability was acceptable (Cronbach’s *α* = 0.82).

### 3.8. Shift‐Based Nursing Skill Mix

Shift‐based nursing skill mix was operationalized as the proportion of registered nurses among registered nurses and nursing assistants working across the day, evening, and night shifts during the previous month. Participants self‐reported the average numbers of registered nurses and nursing assistants working on each shift. These staffing counts were not verified against administrative staffing records. Shift‐based nursing skill mix was calculated as follows:

(1) Shift−based nursing skill mix %=100×RND+RNE+RNNRND+RNE+RNN+NAD+NAE+NAN,

where RN denotes registered nurses, NA denotes nursing assistants, and *D*, *E*, and *N* denote day, evening, and night shifts, respectively. Higher values indicated a greater proportion of registered nurses among the two staffing categories. The alternative ward‐total registered‐nurse proportion was examined only in a supporting sensitivity analysis (see Supporting Table S3).

### 3.9. Statistical Analysis

Before analysis, item ranges, scale scoring, and derived staffing variables were verified; details of data‐quality checks and related sensitivity analyses are provided in Supporting Table S1. Descriptive statistics were used to summarize participant characteristics and study variables. Categorical variables were reported as frequencies and percentages, and continuous variables as means and standard deviations or medians and interquartile ranges, according to their distributions. Percentages in the patient‐safety‐incident‐experience columns of Table 1 were calculated using the corresponding group totals as denominators. Differences in categorical characteristics by incident experience were examined using chi‐squared tests or Fisher’s exact tests when expected cell counts were small. Continuous‐variable comparisons by incident experience were examined using one‐way analysis of variance. Comparisons according to awareness of reporting procedures used Mann–Whitney *U* tests, with rank‐biserial correlations as effect‐size estimates.

**TABLE 1:** Characteristics of study participants according to patient safety incident experience (*N* = 218).

Characteristic	Category	No incident experience (*n* = 89)	Incident experience (*n* = 129)	Total (*N* = 218)	*χ* ^2^ (df)	*p*
		*n* (%)	*n* (%)	*n* (%)		
Gender	Male	6 (6.7)	1 (0.8)	7 (3.2)	—	0.019^a^
	Female	83 (93.3)	128 (99.2)	211 (96.8)		

Age, years	≤ 29	43 (48.3)	35 (27.1)	78 (35.8)	10.74 (2)	0.005
	30–39	34 (38.2)	64 (49.6)	98 (45.0)		
	≥ 40	12 (13.5)	30 (23.3)	42 (19.3)		

Marital status	Single	58 (65.2)	77 (59.7)	135 (61.9)	0.67 (1)	0.413
	Married	31 (34.8)	52 (40.3)	83 (38.1)		

Educational level	Associate	23 (25.8)	40 (31.0)	63 (28.9)	—	0.733^a^
	Bachelor	65 (73.0)	88 (68.2)	153 (70.2)		
	Graduate	1 (1.1)	1 (0.8)	2 (0.9)		

Work experience, years	< 1	5 (5.6)	0 (0.0)	5 (2.3)	—	0.002^a^
	1 to < 5	31 (34.8)	31 (24.0)	62 (28.4)		
	5 to < 10	31 (34.8)	42 (32.6)	73 (33.5)		
	≥ 10	22 (24.7)	56 (43.4)	78 (35.8)		

Number of beds	< 100	32 (36.0)	71 (55.0)	103 (47.2)	7.70 (2)	0.021
	100–149	33 (37.1)	34 (26.4)	67 (30.7)		
	≥ 150	24 (27.0)	24 (18.6)	48 (22.0)		

Awareness of near‐miss and patient safety incident reporting procedures	Yes	75 (84.3)	115 (89.1)	190 (87.2)	1.12 (1)	0.290
	No	14 (15.7)	14 (10.9)	28 (12.8)		

*Note:* Values are *n* (%). Percentages may not total 100 because of rounding. Group differences were tested with Pearson’s *χ* ^2^ test.

Abbreviation: df, degrees of freedom.

^a^Fisher’s exact test (*χ* ^2^ not applicable, shown as —).

Before regression modeling, the distribution of the patient safety nursing activity score was examined using its observed range, empirical quantiles, number of unique values, percentage of exact maximum scores, percentage of scores at or above 4.75, and numbers of observations tied at the selected upper quantiles.

Ordinary least squares (OLS) regression was used to estimate conditional‐mean associations. Standard errors and 95% confidence intervals were calculated using the heteroskedasticity‐consistent covariance matrix estimator type 3 (HC3). Quantile regression models were fitted at the 10th, 25th, 50th, 75th, and 85th percentiles. All six SEIPS‐informed predictors were entered simultaneously: safety control, near‐miss reporting intention, ethical leadership, organizational communication, non‐nursing task performance, and shift‐based nursing skill mix. Shift‐based nursing skill mix was expressed per 10‐percentage‐point increase.

Quantile‐regression uncertainty was estimated using 2000 participant‐level pairs‐bootstrap resamples. For each predictor, a global test evaluated the null hypothesis that its coefficients were equal across the five quantiles:

(2) H0:β0.10=β0.25=β0.50=β0.75=β0.85.

Holm’s method was used to adjust the six predictor‐level heterogeneity‐test *p*‐values. Pointwise significance at an individual quantile was not interpreted as evidence of coefficient heterogeneity unless supported by the global equality test. Model fit was summarized using *R* ^2^ and adjusted *R* ^2^ for OLS regression and pseudo‐*R* ^2^ for quantile regression.

As a bounded‐outcome sensitivity analysis, the activity score was transformed to the 0–1 scale as (*Y* − 1)/4, and a fractional‐logit model was fitted using the same six predictors. The HC3 estimator was used to calculate robust standard errors and 95% confidence intervals, and average marginal effects were rescaled to the original 1–5 outcome metric. Additional sensitivity analyses compared the primary shift‐based skill‐mix measure with the alternative ward‐total registered‐nurse proportion and separate staffing components. All tests were two‐sided, with *p* < 0.05 considered statistically significant. Statistical analyses were conducted using Python version 3.13.5.

### 3.10. Ethical Considerations

This study was conducted in accordance with the Declaration of Helsinki and approved by the Sunchon National University Institutional Review Board (IRB No. 1040173‐202502‐HR‐002‐02). All participants received written study information and provided informed consent before completing the questionnaire.

## 4. Results

### 4.1. Participant Characteristics by Patient Safety Incident Experience

The final analytic sample comprised 218 nurses. Among them, 129 (59.2%) reported one or more patient safety incident experiences, whereas 89 (40.8%) reported none. Most participants were female (*n* = 211, 96.8%). The largest age group was 30–39 years (*n* = 98, 45.0%), followed by ≤ 29 years (*n* = 78, 35.8%) and ≥ 40 years (*n* = 42, 19.3%). Most participants held a bachelor’s degree (*n* = 153, 70.2%), and 78 (35.8%) had at least 10 years of clinical experience. Awareness of near‐miss and patient safety incident reporting procedures was reported by 190 participants (87.2%). Patient safety incident experience differed significantly by gender (Fisher’s exact test, *p* = 0.019), age (*χ* ^2^(2) = 10.74, *p* = 0.005), work experience (Fisher’s exact test, *p* = 0.002), and number of beds (*χ* ^2^(2) = 7.70, *p* = 0.021; Table 1).

### 4.2. Group Differences by Patient Safety Incident Experience

Patient safety nursing activities did not differ significantly between nurses with and without patient safety incident experience. Mean total scores were 4.46 (SD, 0.48) in the no incident experience group and 4.46 (SD, 0.46) in the incident experience group (*F*(1,  216) < 0.01, *p* = 0.996). None of the six patient safety nursing activity subdomains differed significantly between groups. The shift‐based nursing skill mix was 67.3% (SD, 6.5) in the no incident experience group and 66.7% (SD, 6.2) in the incident experience group (*F*(1,  216) = 0.51, *p* = 0.477). Organizational communication was higher in the no incident experience group than in the incident experience group, but the difference was not statistically significant (mean [SD], 3.74 [0.65] vs. 3.57 [0.64]; *F*(1,  216) = 3.86, *p* = 0.051). The remaining person‐, organizational‐, and task‐related factors also did not differ significantly by incident experience (Table 2).

**TABLE 2:** Group comparison of patient safety nursing activities and related factors according to patient safety incident experience (*N* = 218).

Domain	Variable	No incident experience (*n* = 89)	Incident experience (*n* = 129)	Total (*N* = 218)	*F* (1, 216)	*p*
		Mean (SD)	Mean (SD)	Mean (SD)		
Person‐level factors	Safety control	3.59 (0.64)	3.71 (0.59)	3.66 (0.62)	2.13	0.146
	Near‐miss reporting intention	4.03 (0.62)	4.04 (0.71)	4.03 (0.67)	0.01	0.909

Organizational‐level factors	Ethical leadership	4.00 (0.79)	3.86 (0.78)	3.92 (0.78)	1.85	0.175
	Organizational communication	3.74 (0.65)	3.57 (0.64)	3.64 (0.65)	3.86	0.051

Task‐level factors	Non‐nursing task performance	1.15 (0.39)	1.17 (0.42)	1.16 (0.41)	0.20	0.657
	Shift‐based nursing skill mix, %	67.3 (6.5)	66.7 (6.2)	66.9 (6.3)	0.51	0.477

Patient safety nursing activities	Total score	4.46 (0.48)	4.46 (0.46)	4.46 (0.47)	< 0.01	0.996
	Accurate patient identification	4.54 (0.50)	4.58 (0.48)	4.56 (0.49)	0.44	0.506
	Communication among healthcare professionals	4.15 (0.71)	4.07 (0.71)	4.10 (0.71)	0.71	0.400
	High‐alert medication management	4.52 (0.55)	4.63 (0.58)	4.59 (0.57)	1.96	0.163
	Accurate surgery/procedure confirmation	4.46 (0.62)	4.52 (0.57)	4.50 (0.59)	0.41	0.521
	Infection prevention activities	4.57 (0.48)	4.53 (0.51)	4.55 (0.50)	0.38	0.540
	Fall prevention activities	4.58 (0.53)	4.63 (0.58)	4.61 (0.56)	0.40	0.529

*Note:* Group differences were tested with one‐way analysis of variance. Scores range from 1 to 5, except non‐nursing task performance (0–2) and shift‐based nursing skill mix (proportion of registered nurses, %).

Abbreviation: SD, standard deviation.

### 4.3. Distribution of Patient Safety Nursing Activity Scores

Patient safety nursing activity scores were concentrated near the upper boundary. Scores ranged from 3.00 to 5.00, with a mean of 4.46 (SD, 0.47) and a median of 4.54 (IQR, 4.08–4.88). The empirical 75th and 85th percentiles were 4.88 and 4.96, respectively. Thirty participants (13.8%) obtained the exact maximum score of 5.00, and 85 (39.0%) scored 4.75 or higher. The distribution and selected empirical quantiles are shown in Figure 2; detailed ceiling‐effect and tie diagnostics are provided in Supporting Table S2.

**FIGURE 2:**
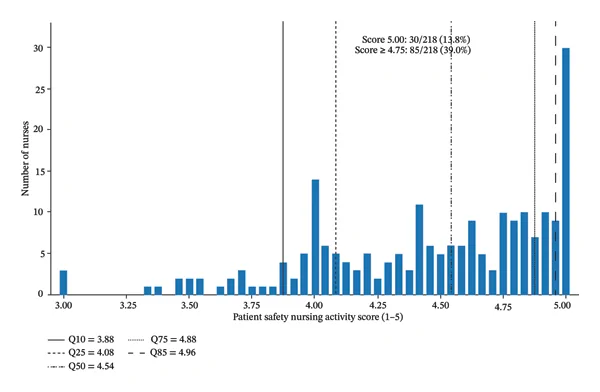
Distribution of patient safety nursing activity scores and selected empirical quantiles among participating nurses (*N* = 218). Note: Bars show the number of nurses at each observed score (scores change in steps of 1/24). Vertical reference lines denote Q10, Q25, Q50, Q75, and Q85 (10th–85th percentiles); the annotations show the numbers of nurses at the maximum score and at scores ≥ 4.75.

### 4.4. Factors Associated With Patient Safety Nursing Activities

The OLS model was statistically significant and explained 34.6% of the variance in patient safety nursing activities (*R* ^2^ = 0.346, adjusted *R* ^2^ = 0.328, *F*(6, 211) = 18.61, *p* < 0.001). Safety control (*B* = 0.172, 95% CI, 0.053–0.291), near‐miss reporting intention (*B* = 0.105, 95% CI, 0.010–0.200), ethical leadership (*B* = 0.139, 95% CI, 0.015–0.264), and non‐nursing task performance (*B* = 0.170, 95% CI, 0.028–0.313) were positively associated with patient safety nursing activities. Organizational communication (*B* = 0.054, 95% CI, −0.072–0.180) and shift‐based nursing skill mix per 10‐percentage‐point increase (*B* = −0.074, 95% CI, −0.170 to 0.021) were not statistically significant.

In the quantile models, pointwise bootstrap confidence intervals excluded zero for safety control at Q10, Q25, and Q50; near‐miss reporting intention at Q25; ethical leadership at Q25 and Q50; non‐nursing task performance at Q50; and shift‐based nursing skill mix at Q50. These pointwise findings were not interpreted as evidence that coefficients differed across quantiles.

In the formal coefficient‐equality tests, safety control showed nominal evidence of heterogeneity across quantiles (global *p* = 0.010), but this did not remain statistically significant after Holm adjustment (*p* = 0.060). None of the other predictors showed significant adjusted coefficient heterogeneity; Holm‐adjusted *p*‐values ranged from 0.490 to > 0.999. The pseudo‐*R* ^2^ values were 0.259, 0.270, 0.244, 0.155, and 0.117 at Q10, Q25, Q50, Q75, and Q85, respectively. Point estimates and equality tests are presented in Table 3; coefficient trajectories with bootstrap 95% confidence intervals are shown in Figure 3 and Supporting Table S4.

**TABLE 3:** Ordinary least squares and quantile‐regression estimates for factors associated with patient safety nursing activities (*N* = 218).

Predictor	OLS	Quantile regression, *B*					Global *p*	Holm *p*
	*B* (95% CI)	Q10	Q25	Q50	Q75	Q85		
*Person-level factors*								
Safety control	0.172 (0.053–0.291)	0.233^a^	0.266^a^	0.184^a^	0.036	0.026	0.010	0.060
Near‐miss reporting intention	0.105 (0.010–0.200)	0.096	0.142^a^	0.079	0.044	0.051	0.730	> 0.999

*Organizational-level factors*								
Ethical leadership	0.139 (0.015–0.264)	0.118	0.151^a^	0.156^a^	0.121	0.019	0.098	0.490
Organizational communication	0.054 (−0.072–0.180)	0.083	0.026	0.105	0.067	0.068	0.883	> 0.999

*Task-level factors*								
Non‐nursing task performance	0.170 (0.028–0.313)	0.245	0.187	0.246^a^	0.086	0.093	0.397	> 0.999
Shift‐based nursing skill mix, per 10 percentage points	−0.074 (−0.170 to 0.021)	−0.074	−0.106	−0.157^a^	−0.027	−0.055	0.220	0.878
*R* ^2^ (OLS) or pseudo‐*R* ^2^ (quantile)	0.346	0.259	0.270	0.244	0.155	0.117		

*Note. B*, unstandardized regression coefficient (change in the patient safety nursing activity score per 1‐point increase in each predictor, or per 10‐percentage‐point increase in shift‐based nursing skill mix); Q10–Q85, 10th–85th conditional quantiles. OLS 95% CIs were calculated with the heteroskedasticity‐consistent covariance matrix estimator type 3 (HC3); adjusted *R* ^2^ = 0.328, *F* (6, 211) = 18.61, *p* < 0.001.

Abbreviations: CI, confidence interval; OLS, ordinary least squares.

^a^95% CI excludes zero (2000 participant‐level pairs‐bootstrap resamples); quantile‐specific 95% CIs are shown in Figure 3 and Supporting Table S4. Global *p*, test of equality of each coefficient across the five quantiles; Holm *p*, Global *p* adjusted for six predictors with the Holm method.

**FIGURE 3:**
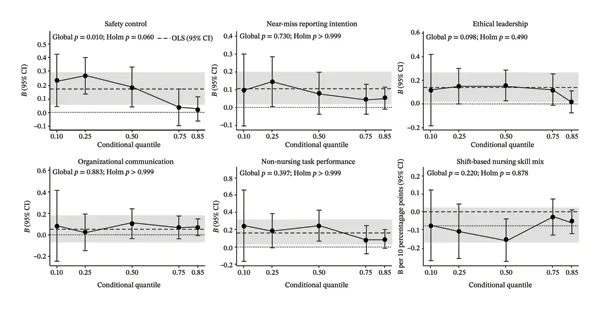
Quantile‐specific coefficient estimates with participant‐level pairs‐bootstrap 95% confidence intervals and OLS reference estimates. Note: Points and error bars show quantile‐regression coefficients (*B*) and 95% confidence intervals from 2000 participant‐level pairs‐bootstrap resamples; the dashed line and gray band show the ordinary least squares (OLS) estimate and its HC3 95% confidence interval. Global p and Holm p are defined in Table 3. Shift‐based nursing skill mix is expressed per 10‐percentage‐point increase.

### 4.5. Group Differences by Awareness of Reporting Procedures

Nurses who reported awareness of near‐miss and patient safety incident reporting procedures had higher near‐miss reporting intention than those without such awareness (median, 4.00 vs. 3.75; *U* = 3, 506.0, *p* = 0.006, rank‐biserial *r* = 0.32). Patient safety nursing activities were also higher in the aware group (median, 4.58; IQR, 4.21–4.88) than in the not‐aware group (median, 4.08; IQR, 3.82–4.75; *U* = 3, 469.0, *p* = 0.009, *r* = 0.30). Safety control, ethical leadership, organizational communication, non‐nursing task performance, and shift‐based nursing skill mix did not differ significantly between groups (Table 4).

**TABLE 4:** Comparison of patient safety factors and nursing activities according to awareness of near‐miss and patient safety incident reporting procedures (*N* = 218).

Variable	Not aware (*n* = 28)	Aware (*n* = 190)	*U*	*p*	*r*
	Median (IQR)	Median (IQR)			
Safety control	3.57 (3.00–3.89)	3.71 (3.29–4.00)	3259.5	0.053	0.23
Near‐miss reporting intention	3.75 (3.44–4.00)	4.00 (3.75–4.75)	3506.0	0.006	0.32
Ethical leadership	3.65 (3.08–4.30)	4.00 (3.50–4.50)	3091.5	0.164	0.16
Organizational communication	3.50 (3.07–4.00)	3.61 (3.22–4.00)	2926.5	0.393	0.10
Non‐nursing task performance	1.11 (0.75–1.33)	1.11 (0.89–1.44)	2921.0	0.401	0.10
Shift‐based nursing skill mix, %	70.00 (61.15–73.68)	67.71 (63.16–71.43)	2518.5	0.650	−0.05
Patient safety nursing activities	4.08 (3.82–4.75)	4.58 (4.21–4.88)	3469.0	0.009	0.30

*Note:* Values are median (IQR), with the IQR presented as the 25th–75th percentiles. Aware and not aware refer to awareness of near‐miss and patient safety incident reporting procedures, respectively. Groups were compared with the Mann–Whitney *U* test. *r*, rank‐biserial correlation (effect size); positive values indicate higher scores in the aware group. Nursing skill mix is the shift‐based registered‐nurse proportion. IQR, interquartile range.

### 4.6. Bounded‐Outcome and Staffing Sensitivity Analyses

The fractional‐logit sensitivity analysis produced a pattern similar to the OLS analysis. On the original 1–5 scale, average marginal effects were positive for safety control (AME = 0.172, 95% CI, 0.060–0.285, *p* = 0.003), near‐miss reporting intention (AME = 0.125, 95% CI, 0.044–0.206, *p* = 0.003), ethical leadership (AME = 0.120, 95% CI, 0.009–0.232, *p* = 0.035), and non‐nursing task performance (AME = 0.198, 95% CI, 0.056–0.339, *p* = 0.006). Organizational communication was not significant (AME = 0.078, 95% CI, −0.043 to 0.199, *p* = 0.206), and shift‐based nursing skill mix was also not significant (AME = −0.079 per 10‐percentage‐point increase, 95% CI, −0.171 to 0.014, *p* = 0.095; Table 5).

**TABLE 5:** Fractional‐logit sensitivity analysis of factors associated with patient safety nursing activities (*N* = 218).

Predictor	Logit coefficient (95% CI)	AME (95% CI)^a^	*p* ^b^
*Person-level factors*			
Safety control	0.387 (0.140–0.635)	0.172 (0.060–0.285)	0.003
Near‐miss reporting intention	0.281 (0.096–0.466)	0.125 (0.044–0.206)	0.003

*Organizational-level factors*			
Ethical leadership	0.271 (0.023–0.519)	0.120 (0.009–0.232)	0.035
Organizational communication	0.176 (−0.099–0.451)	0.078 (−0.043–0.199)	0.206

*Task-level factors*			
Non‐nursing task performance	0.445 (0.119–0.771)	0.198 (0.056–0.339)	0.006
Shift‐based nursing skill mix, per 10 percentage points	−0.177 (−0.388 to 0.033)	−0.079 (−0.171 to 0.014)	0.095

*Note:* The outcome was rescaled to (score − 1)/4 for fractional‐logit regression. Predictors are expressed per 1‐point increase, except shift‐based nursing skill mix (per 10‐percentage‐point increase). Robust standard errors and 95% CIs were calculated with the heteroskedasticity‐consistent covariance matrix estimator type 3 (HC3).

^a^AMEs were rescaled to the original 1–5 outcome metric.

^b^ *p* value for the AME. AME, average marginal effect; CI, confidence interval.

In the staffing sensitivity analysis, the alternative ward‐total registered‐nurse proportion showed a negative association, whereas the primary shift‐based ratio did not. The estimated association therefore differed according to the operational definition of nursing skill mix (Supporting Table S3).

## 5. Discussion

This study examined work‐system factors associated with patient safety nursing activities among nurses working in integrated nursing care service wards of nonaccredited small‐ and medium‐sized hospitals. Patient safety nursing activity scores were high and concentrated near the upper boundary. In the conditional‐mean analyses, safety control, near‐miss reporting intention, ethical leadership, and non‐nursing task performance were positively associated with the outcome, whereas organizational communication and shift‐based nursing skill mix were not. The fractional‐logit sensitivity analysis showed a similar overall pattern. Among the quantile‐regression findings, safety control showed the clearest exploratory distributional pattern, with larger coefficient estimates at lower‐to‐middle conditional quantiles than near the upper boundary. Taken together, the findings demonstrate that patient safety nursing activities are associated with selected factors across multiple components of the work system.

Safety control was positively associated with patient safety nursing activities in the OLS and fractional‐logit analyses and showed the clearest exploratory distributional pattern. However, the cross‐quantile variation did not remain statistically significant after Holm adjustment; the pattern therefore represents a descriptive distributional signal rather than a confirmed difference between quantiles. The overall positive association is consistent with previous evidence linking safety control to nurses’ patient safety activities [8]. Near‐miss reporting intention was also positively associated with patient safety nursing activities, and nurses who were aware of reporting procedures had higher reporting intention and patient safety nursing activity scores. These findings reinforce the relevance of accessible reporting procedures and organizational learning from near misses [1–3, 7].

Ethical leadership was positively associated with patient safety nursing activities in both models, consistent with previous research linking ethical and safety‐oriented leadership to patient safety culture and safety‐related behavior [19, 21]. In contrast, organizational communication was not independently associated with the outcome. This differs from studies reporting positive relationships involving communication and teamwork. The difference may relate to the broad formal and informal communication construct assessed in this study, whereas safety‐specific communication, speaking up, and clinical handoffs may be more directly related to patient safety practice [23]. Future longitudinal or mediation studies could clarify whether organizational communication is indirectly related to patient safety nursing activities through broader organizational and team processes.

At the task level, non‐nursing task performance was positively associated with patient safety nursing activities in the OLS and fractional‐logit analyses. This finding differs from previous studies linking non‐nursing work with reduced direct‐care time, missed nursing care, and adverse outcomes [5, 6, 24]. The instrument used in this study measured the frequency of nine non‐nursing tasks rather than the time spent on these tasks or their workload burden. Accordingly, the observed coefficient represents an association between the reported frequencies of non‐nursing tasks and patient safety nursing activities rather than an effect of non‐nursing work on safety practice. Accordingly, the positive association observed in this study should be interpreted in relation to the reported frequency of non‐nursing task performance rather than the workload or time burden associated with those tasks.

Shift‐based nursing skill mix was not independently associated with patient safety nursing activities. This finding differs from previous studies linking higher registered‐nurse staffing levels or registered‐nurse proportions with less missed care and better patient outcomes [3, 25–27]. In the sensitivity analysis, the alternative ward‐total registered‐nurse proportion showed a negative association, whereas the primary shift‐based measure did not (Supporting Table S3). This difference indicates that the observed association was sensitive to the level and time frame used to operationalize nursing skill mix. Future studies should use verified staffing records and compare shift‐level and ward‐level measures to clarify how the operational definition of nursing skill mix relates to patient safety nursing activities.

### 5.1. Implications for Nursing Management

For nursing management, the positive associations of safety control, near‐miss reporting intention, and ethical leadership with patient safety nursing activities highlight these factors as relevant areas for organizational attention. Clear reporting procedures and leadership practices that support patient safety may therefore be considered within ward‐level safety management.

The findings also highlight the importance of assessing task allocation and staffing in nursing management. Task allocation should be considered in relation to the content and demands of nurses’ work, while staffing composition should be evaluated using multiple indicators rather than a single skill‐mix ratio.

### 5.2. Limitations

Several limitations warrant consideration. First, the cross‐sectional, self‐report design precludes causal inference and may have introduced recall, social desirability, and common‐method bias. Second, patient safety nursing activity scores were concentrated near the upper boundary, resulting in limited score variability at the 75th and 85th percentiles. Third, shift‐based nursing skill mix was calculated from participants’ self‐reported average staffing across day, evening, and night shifts; therefore, the staffing estimates were susceptible to recall and reporting error. Fourth, the final models did not incorporate workload, patient acuity, nurse‐to‐patient ratios, missed nursing care, or broader support‐staff deployment. Future studies should examine these care‐demand and workforce characteristics together to clarify the associations of non‐nursing task performance and staffing with patient safety nursing activities. Finally, convenience sampling from 10 nonaccredited small‐ and medium‐sized hospitals in two regions of South Korea may limit generalizability to accredited hospitals, other hospital types, and other regions.

## 6. Conclusions

Patient safety nursing activities in integrated nursing care service wards were associated with nurses’ safety control and near‐miss reporting intention, perceived ethical leadership, and non‐nursing task performance. Safety control showed the clearest exploratory distributional pattern, with larger coefficient estimates at lower‐to‐middle conditional quantiles than near the upper boundary. Considering both the average level and the underlying score distribution provides nursing management with a more complete understanding of variation in patient safety nursing practice than considering average scores alone.

## Funding

This research received no specific grant from any funding agency in the public, commercial, or not‐for‐profit sectors.

## Disclosure

This paper is based on and expanded from the first author’s master’s thesis.

## Conflicts of Interest

The authors declare no conflicts of interest.

## Supporting Information

Additional supporting information can be found online in the Supporting Information section.

## Supporting information


**Supporting Information 1** The following supporting information is available online: Supporting Table S1. Variable construction, data‐quality decisions, and data‐handling sensitivity analyses. Panel A explains how each study variable was operationalized, including the primary shift‐based and alternative ward‐total nursing skill mix measures. It also reports the data‐audit findings, how they were handled, and why cluster‐aware analyses could not be performed. Panel B compares HC3‐robust OLS estimates from the primary analysis (*N* = 218) with a complete‐case analysis for organizational communication (*n* = 217). Supporting Table S2. Detailed distribution, ceiling‐effect, and tie diagnostics for patient safety nursing activities (*N* = 218). It reports descriptive statistics, selected percentiles (Q10–Q85), the number of nurses at or near the maximum score, tied observations at the upper quantiles, skewness, and excess kurtosis. These results support the use of quantile regression and the interpretation of upper‐tail estimates. Supporting Table S3. Sensitivity of staffing associations to the operational definition of nursing skill mix and to how staffing components were specified (*N* = 218). It compares OLS coefficients and fractional‐logit average marginal effects, with 95% confidence intervals, across four sets of models: the primary shift‐based ratio, the alternative ward‐total ratio, models adding total staffing, and models with separate RN and nursing‐assistant counts. Supporting Table S4. Quantile‐regression coefficients with 95% confidence intervals for factors associated with patient safety nursing activities (*N* = 218). It gives the 10th, 25th, 50th, 75th, and 85th quantile estimates summarized in Table 3 and plotted in Figure 3, with confidence intervals from 2000 participant‐level pairs‐bootstrap resamples.

**Supporting Information 2** STROBE_Checklist_QR_0610.

## Data Availability

The data that support the findings of this study are available from the corresponding author upon reasonable request. The data are not publicly available due to institutional ethics approval requirements and privacy restrictions.
