# Supplementary material for: Work‐System Factors Associated With Patient Safety Nursing Activities in Integrated Nursing Care Service Wards of Nonaccredited Small‐ and Medium‐Sized Hospitals in Korea: A Cross‐Sectional Study With Exploratory Quantile Regression
Source: J Nurs Manag. 2026 Nov 9;2026:9403660. doi: 10.1155/jonm/9403660 (PMC13748806; doi:10.1155/jonm/9403660)
Supplement: Supplementary file 1 [file JONM-2026-9403660-s001.docx]

**Supplementary Material**

**Supplementary Table S1.** Variable construction, data-quality decisions, and data-handling sensitivity analyses

*Panel A. Variable construction and data-audit decisions*

| **Construct or data element** | **Operationalization in the primary reanalysis** | **Role in analysis** | **Data-audit finding and handling** |
| --- | --- | --- | --- |
| Patient safety nursing activities | Mean of 24 items scored 1–5 | Primary outcome | No missing or out-of-range item values were identified. |
| Safety control | Mean of 7 items scored 1–5 | Primary predictor | No missing or out-of-range item values were identified. |
| Near-miss reporting intention | Mean of 4 items scored 1–5 | Primary predictor | No missing or out-of-range item values were identified. |
| Ethical leadership | Mean of 10 items scored 1–5 | Primary predictor | No missing or out-of-range item values were identified. |
| Organizational communication | Available-item mean of 23 items scored 1–5 | Primary predictor | One item was blank for one participant. The primary analysis used the available-item mean; a complete-case sensitivity analysis used n = 217. |
| Non-nursing task performance | Mean of 9 items scored 0–2 | Primary predictor | Item ranges and coding were verified against the source questionnaire before analysis. |
| Shift-based nursing skill mix (primary; Nursing Skill Mix 2) | 100 × sum of average day, evening, and night registered-nurse counts / sum of registered-nurse and nursing-assistant counts across the same shifts | Primary staffing predictor | Calculated from participants’ self-reported average staffing actually working across shifts during the previous month. Recalculation matched the workbook value. |
| Ward-total nursing skill mix (alternative; Nursing Skill Mix 1) | 100 × total ward registered nurses / total ward registered nurses and nursing assistants | Supplementary sensitivity analysis only | Calculated from participant-reported ward-total headcounts. It was not entered in the primary model. |
| Hospital or ward cluster identifier | Not available in the analysis workbook | Not estimable | Cluster-aware standard errors, multilevel analysis, and leave-one-hospital-out analysis could not be performed. |

*Panel B. OLS sensitivity to the identified data-handling decisions*

| **Analysis** | **N** | **R²** | **B** | | | | |
| --- | --- | --- | --- | --- | --- | --- | --- |
|  |  |  | **Safety control** | **Near-miss reporting intention** | **Ethical leadership** | **Non-nursing task performance** | **Shift-based nursing skill mix^a^** |
| Primary handling | 218 | 0.346 | 0.172 | 0.105 | 0.139 | 0.170 | −0.074 |
| Complete-case organizational communication | 217 | 0.345 | 0.172 | 0.105 | 0.140 | 0.171 | −0.074 |

*Note.* Panel B entries are unstandardized OLS coefficients (B) with heteroskedasticity-consistent covariance matrix estimator type 3 (HC3) standard errors. ^a^Per 10-percentage-point increase. OLS, ordinary least squares.

**Supplementary Table S2.** Detailed distribution, ceiling-effect, and tie diagnostics for patient safety nursing activities (N = 218)

| **Statistic** | **Value** |
| --- | --- |
| Mean (SD) | 4.46 (0.47) |
| Median (IQR) | 4.54 (4.08–4.88) |
| Observed range | 3.00–5.00 |
| 10th percentile (Q10) | 3.88 |
| 25th percentile (Q25) | 4.08 |
| 50th percentile (Q50) | 4.54 |
| 75th percentile (Q75) | 4.88 |
| 85th percentile (Q85) | 4.96 |
| Unique observed mean scores, n | 40 |
| Exact maximum score (5.00), n (%) | 30 (13.8) |
| Score ≥4.75, n (%) | 85 (39.0) |
| Tied observations at the Q75 score, n | 7 |
| Tied observations at the Q85 score, n | 9 |
| Skewness | −0.80 |
| Excess kurtosis | 0.09 |

*Note.* The outcome was the mean of 24 items scored 1–5 and therefore changed in increments of 1/24 (approximately 0.042). Q85 was one item-response increment below the maximum, indicating limited room for variation in the upper tail. IQR, interquartile range (25th–75th percentiles); SD, standard deviation.

**Supplementary Table S3.** Sensitivity of staffing associations to the operational definition of nursing skill mix and staffing-component specification (N = 218)

| **Specification** | **Staffing term** | **OLS** | | **Fractional logit** | |
| --- | --- | --- | --- | --- | --- |
|  |  | **B (95% CI)** | **p** | **AME (95% CI)** | **p** |
| Primary: shift-based ratio | Shift-based skill mix (per 10 pp) | −0.074 (−0.170 to 0.021) | 0.126 | −0.079 (−0.171 to 0.014) | 0.095 |
| Shift ratio + total shift staffing | Shift-based skill mix (per 10 pp) | −0.074 (−0.170 to 0.021) | 0.126 | −0.080 (−0.172 to 0.013) | 0.090 |
|  | Total shift staffing (per 5 staff) | 0.000 (−0.077 to 0.077) | >0.999 | −0.006 (−0.075 to 0.063) | 0.867 |
| Shift components | Shift RN count (per 5 staff) | −0.084 (−0.199 to 0.031) | 0.150 | −0.097 (−0.205 to 0.011) | 0.079 |
|  | Shift nursing-assistant count (per 5 staff) | 0.179 (−0.025 to 0.383) | 0.085 | 0.186 (−0.008 to 0.381) | 0.061 |
| Alternative: ward-total ratio | Ward-total skill mix (per 10 pp) | −0.127 (−0.222 to −0.033) | 0.008 | −0.135 (−0.219 to −0.051) | 0.002 |
| Ward ratio + total ward staffing | Ward-total skill mix (per 10 pp) | −0.128 (−0.225 to −0.031) | 0.010 | −0.135 (−0.220 to −0.050) | 0.002 |
|  | Total ward staffing (per 10 staff) | 0.008 (−0.085 to 0.101) | 0.869 | −0.001 (−0.083 to 0.081) | 0.974 |
| Ward-total components | Ward RN count (per 5 staff) | −0.065 (−0.124 to −0.006) | 0.032 | −0.075 (−0.127 to −0.023) | 0.005 |
|  | Ward nursing-assistant count (per 5 staff) | 0.172 (0.026 to 0.317) | 0.021 | 0.184 (0.052 to 0.317) | 0.006 |

*Note.* All specifications were simultaneously adjusted for safety control, near-miss reporting intention, ethical leadership, organizational communication, and non-nursing task performance. The shift-based registered-nurse proportion was the primary staffing predictor; the ward-total registered-nurse proportion was evaluated only as an alternative sensitivity specification. Staffing data were derived from participants’ self-reported current ward and average shift counts. OLS 95% CIs were calculated with the heteroskedasticity-consistent covariance matrix estimator type 3 (HC3). Fractional-logit AMEs were rescaled to the original 1–5 outcome metric. Because hospital and ward identifiers were unavailable, cluster-aware inference could not be estimated. AME, average marginal effect; B, unstandardized regression coefficient; CI, confidence interval; OLS, ordinary least squares; pp, percentage points; RN, registered nurse.

**Supplementary Table S4.** Quantile-regression coefficients with 95% confidence intervals for factors associated with patient safety nursing activities (N = 218)

| **Predictor** | **B (95% CI)** | | | | |
| --- | --- | --- | --- | --- | --- |
|  | **Q10** | **Q25** | **Q50** | **Q75** | **Q85** |
| *Person-level factors* | | | | | |
| Safety control | 0.233 (0.044 to 0.422) | 0.266 (0.133 to 0.400) | 0.184 (0.039 to 0.329) | 0.036 (−0.097 to 0.169) | 0.026 (−0.060 to 0.112) |
| Near-miss reporting intention | 0.096 (−0.105 to 0.297) | 0.142 (0.003 to 0.282) | 0.079 (−0.038 to 0.196) | 0.044 (−0.038 to 0.125) | 0.051 (−0.008 to 0.110) |
| *Organizational-level factors* | | | | | |
| Ethical leadership | 0.118 (−0.184 to 0.420) | 0.151 (0.002 to 0.299) | 0.156 (0.027 to 0.285) | 0.121 (−0.012 to 0.255) | 0.019 (−0.073 to 0.111) |
| Organizational communication | 0.083 (−0.250 to 0.416) | 0.026 (−0.142 to 0.194) | 0.105 (−0.034 to 0.244) | 0.067 (−0.038 to 0.171) | 0.068 (−0.012 to 0.148) |
| *Task-level factors* | | | | | |
| Non-nursing task performance | 0.245 (−0.167 to 0.658) | 0.187 (−0.007 to 0.380) | 0.246 (0.074 to 0.418) | 0.086 (−0.072 to 0.245) | 0.093 (−0.011 to 0.197) |
| Shift-based nursing skill mix, per 10 percentage points | −0.074 (−0.270 to 0.121) | −0.106 (−0.256 to 0.045) | −0.157 (−0.275 to −0.039) | −0.027 (−0.127 to 0.073) | −0.055 (−0.121 to 0.011) |

*Note.* Confidence intervals were obtained from 2,000 participant-level pairs-bootstrap resamples. Predictors are expressed per 1-point increase, except shift-based nursing skill mix (per 10-percentage-point increase). The corresponding OLS estimates, Global p, and Holm p are shown in Table 3, and the estimates are plotted in Figure 3. B, unstandardized regression coefficient; CI, confidence interval; OLS, ordinary least squares; Q10–Q85, 10th–85th conditional quantiles.
