# Supplementary material for: Work‐System Factors Associated With Patient Safety Nursing Activities in Integrated Nursing Care Service Wards of Nonaccredited Small‐ and Medium‐Sized Hospitals in Korea: A Cross‐Sectional Study With Exploratory Quantile Regression
Source: J Nurs Manag. 2026 Nov 9;2026:9403660. doi: 10.1155/jonm/9403660 (PMC13748806; doi:10.1155/jonm/9403660)
Supplement: Supplementary file 2 [file JONM-2026-9403660-s002.docx]

**STROBE Statement—Checklist of items that should be included in reports of cross-sectional studies**

*Manuscript: “Factors associated with patient safety nursing activities among nurses in non-accredited small and medium-sized hospitals: A quantile regression study in Korea” — submitted to Journal of Clinical Nursing*

| **Item No** | **Recommendation** | **Page no. & location in manuscript** |
| --- | --- | --- |
| **Title and abstract** | | |
| **1** | (a) Indicate the study’s design with a commonly used term in the title or the abstract  (b) Provide in the abstract an informative and balanced summary of what was done and what was found | **(a) p. 1; p. 2.** Title (“A quantile regression study”); Abstract – Design: “Cross-sectional survey study”  **(b) pp. 2–3.** Structured abstract |
| **Introduction** | | |
| **2** | Background/rationale: Explain the scientific background and rationale for the investigation being reported | **pp. 4–6.** Introduction and Background sections |
| **3** | Objectives: State specific objectives, including any prespecified hypotheses | **p. 6.** Final paragraph of Background (three numbered aims, incl. comparison across the 10th–85th percentiles) |
| **Methods** | | |
| **4** | Study design: Present key elements of study design early in the paper | **p. 7.** Method – Design and Participants (cross-sectional; STROBE-guided) |
| **5** | Setting: Describe the setting, locations, and relevant dates, including periods of recruitment, exposure, follow-up, and data collection | **pp. 7–8.** Design and Participants (10 non-accredited hospitals ≤200 beds, integrated nursing care wards, two regions); Data Collection (March–April 2025) |
| **6** | (a) Give the eligibility criteria, and the sources and methods of selection of participants | **p. 7.** Design and Participants (inclusion: RN with ≥6 months direct ward care; exclusion: managerial/administrative roles without patient contact; convenience recruitment from 10 hospitals) |
| **7** | Variables: Clearly define all outcomes, exposures, predictors, potential confounders, and effect modifiers. Give diagnostic criteria, if applicable | **pp. 8–12.** Measures (outcome and SEIPS person-/organisational-/task-level predictors); Figure 1 conceptual framework, p. 5 |
| **8*** | Data sources/measurement: For each variable of interest, give sources of data and details of methods of assessment (measurement). Describe comparability of assessment methods if there is more than one group | **pp. 8–12.** Measures (each validated instrument with items, scaling, Cronbach’s α); Data Collection (self-administered online/paper questionnaire) |
| **9** | Bias: Describe any efforts to address potential sources of bias | **pp. 8, 12, 22.** Data Collection (anonymous sealed envelopes, password-protected database); Statistical Analysis (VIF; awareness variable excluded to avoid overadjustment); Limitations (self-report/recall bias, common-method variance) |
| **10** | Study size: Explain how the study size was arrived at | **p. 7.** Design and Participants (G*Power 3.1.9.7: f²=0.15, α=.05, power=.90, 6 predictors → minimum 123; final N=218) |
| **11** | Quantitative variables: Explain how quantitative variables were handled in the analyses. If applicable, describe which groupings were chosen and why | **pp. 8–12.** Measures (5-point Likert scales; skill mix as continuous %; categorical demographics); Statistical Analysis (outcome modelled at 5 prespecified percentiles) |
| **12** | (a) Describe all statistical methods, including those used to control for confounding  (b) Describe any methods used to examine subgroups and interactions  (c) Explain how missing data were addressed  (d) If applicable, describe analytical methods taking account of sampling strategy  (e) Describe any sensitivity analyses | **(a) p. 12.** Descriptives; χ²/Fisher’s exact; one-way ANOVA; Mann–Whitney U; OLS and quantile regression (R v4.6.0)  **(b) pp. 12–16.** Group comparisons by incident experience and by awareness of reporting procedures; quantile-specific estimates  **(c) p. 7.** 6 incomplete responses excluded; complete-case analysis  **(d) p. 22.** Limitations note ward/hospital clustering not modelled  **(e) p. 12.** Rationale for 85th percentile as upper bound given ceiling effect |
| **Results** | | |
| **13*** | (a) Report numbers of individuals at each stage—e.g. numbers potentially eligible, examined for eligibility, confirmed eligible, included in the study, completing follow-up, and analysed  (b) Give reasons for non-participation at each stage  (c) Consider use of a flow diagram | **(a) pp. 7, 14.** 224 invited and returned, 218 analysed; 97.3% analytic response rate  **(b) p. 7.** 6 questionnaires excluded for incomplete responses  **(c)** Not included – flow diagram can be added on editor request |
| **14*** | (a) Give characteristics of study participants (e.g. demographic, clinical, social) and information on exposures and potential confounders  (b) Indicate number of participants with missing data for each variable of interest | **(a) p. 14; pp. 34, 36.** Participant Characteristics; Tables 1 and 2  **(b) pp. 7, 14.** Complete cases only; analytic N=218 after excluding 6 incomplete responses |
| **15*** | Outcome data: Report numbers of outcome events or summary measures | **p. 15; pp. 36, 40.** Group Differences; Tables 2 and 4 (means ± SD, medians/IQR) |
| **16** | (a) Give unadjusted estimates and, if applicable, confounder-adjusted estimates and their precision (e.g. 95% CI). Make clear which confounders were adjusted for and why they were included  (b) Report category boundaries when continuous variables were categorized  (c) If relevant, consider translating estimates of relative risk into absolute risk for a meaningful time period | **(a) p. 15; p. 38; p. 16.** Factors Associated With Patient Safety Nursing Activities; Table 3 (OLS B and quantile coefficients with SE, R²/pseudo-R²); Figure 2  **(b) p. 34.** Table 1 (age, work experience, bed-number categories)  **(c)** Not applicable – no relative-risk estimates in this design |
| **17** | Other analyses: Report other analyses done—e.g. analyses of subgroups and interactions, and sensitivity analyses | **p. 16; p. 40.** Group Differences According to Awareness of Reporting Procedures; Table 4 (Mann–Whitney U, rank-biserial effect sizes); Tables 1–2 by incident experience |
| **Discussion** | | |
| **18** | Key results: Summarise key results with reference to study objectives | **pp. 17, 23.** Discussion opening; Conclusions |
| **19** | Limitations: Discuss limitations of the study, taking into account sources of potential bias or imprecision. Discuss both direction and magnitude of any potential bias | **pp. 22–23.** Strengths and Limitations of the Work |
| **20** | Interpretation: Give a cautious overall interpretation of results considering objectives, limitations, multiplicity of analyses, results from similar studies, and other relevant evidence | **pp. 17–22.** Discussion (Person-, Organisational-, Task-level subsections; associations interpreted as non-causal) |
| **21** | Generalisability: Discuss the generalisability (external validity) of the study results | **pp. 22–23.** Strengths and Limitations (generalisability limited to non-accredited small/medium hospitals) |
| **Other information** | | |
| **22** | Funding: Give the source of funding and the role of the funders for the present study and, if applicable, for the original study on which the present article is based | **p. 25.** Funding (None); Acknowledgements (based on first author’s Master’s thesis); Conflicts of Interest (none declared) |

**Give such information separately for exposed and unexposed groups where relevant.*

Note: An Explanation and Elaboration article discusses each checklist item and gives methodological background and examples of transparent reporting. The STROBE checklist is best used in conjunction with this article (freely available at www.strobe-statement.org).

*Page numbers correspond to the manuscript file QR_Manuscript_0610_2026_Final.docx (43 pp.). If repagination changes the layout, verify before submission.*
